# Investigating Deep Stock Market Forecasting with Sentiment Analysis

**DOI:** 10.3390/e25020219

**Published:** 2023-01-23

**Authors:** Charalampos M. Liapis, Aikaterini Karanikola, Sotiris Kotsiantis

**Affiliations:** Department of Mathematics, University of Patras, 26504 Patras, Greece

**Keywords:** time series forecasting, deep learning, financial time series, sentiment analysis, financial BERT, multivariate, multi-step, regression, Twitter

## Abstract

When forecasting financial time series, incorporating relevant sentiment analysis data into the feature space is a common assumption to increase the capacities of the model. In addition, deep learning architectures and state-of-the-art schemes are increasingly used due to their efficiency. This work compares state-of-the-art methods in financial time series forecasting incorporating sentiment analysis. Through an extensive experimental process, 67 different feature setups consisting of stock closing prices and sentiment scores were tested on a variety of different datasets and metrics. In total, 30 state-of-the-art algorithmic schemes were used over two case studies: one comparing methods and one comparing input feature setups. The aggregated results indicate, on the one hand, the prevalence of a proposed method and, on the other, a conditional improvement in model efficiency after the incorporation of sentiment setups in certain forecast time frames.

## 1. Introduction

Somewhere in the course of history, the human species’ need for knowledge of possible future outcomes of various events emerged. Associative norms were thus constructed between decision-making and observed data that were influenced by theoretical biases that had been inductively established on the basis of such observations. Protoscience was formed. Or not?

Even if this hypothetical description of human initiation into scientific capacities is naive or even unfounded, the bottom line is that the human species partly operates on the basis of predictions. Observing time-evolving phenomena and questioning their structure in the direction of an understanding that will derive predictions about their projected future behavior constitutes an inherent part of post-primitive human history. In response to this self-referential demand and assuming that the authors are post-primitive individuals, the core of the present work is about predicting sequential and time-dependent phenomena. This domain is called time series forecasting. Time series forecasting is, in broad terms, the process of using a model to predict future values of variables that characterize a phenomenon based on historical data. A time series is a set of time-dependent observations sampled at specific points in time. The sampling rate depends on the nature of the problem. Moreover, depending on the number of variables describing the sequentially recorded observations, a distinction is made between univariate and multivariate time series. Since there is a wide range of time-evolving problems, the field is quite relevant in modern times, with an increasing demand for model accuracy and robustness.

In addition, there are phenomena, the mathematical formalism of which is represented by time series with values which are also sub-determined by the given composition of a society of individuals. This means that the attitudes of such individuals, as they nonetheless form within the whole, are somewhat informative about aspects of the phenomenon in question. It is natural, given human nature and the consequent conceptual treatment of the world as part of it, that these attitudes are articulated somewhere linguistically. Therefore, a hypothesis on which mathematical quantifications of the attitudes of which such linguistic representations that are signs are possible could, if valid, describe a framework for improving the modeling of the phenomena in question. For example, specific economic figures can be points in a context, the elements of which are partially shaped by what is said about them. Accordingly, it can be argued that a line of research that would investigate whether stock closing prices can be modeled in terms of their future fluctuations using relevant linguistic data collected from social networks is valid.

Thus, in this work, the incorporation of sentiment analysis in stock market forecasting is investigated. In particular, a large number of state-of-the-art methods are put under an experimental framework that includes multiple configurations of input features that incorporate quantified values of sentiment attitudes in the form of time series. These time series consist of sentiment scores extracted from Twitter using three different sentiment analysis methods. Regarding prediction methods, there are schemes that come from both the field of statistics and machine learning. Within the machine learning domain, deep learning and other state-of-the-art methods are currently in use, dominating research. Here, a large number of such widely used state-of-the-art models were benchmarked in terms of performance. Moreover, various sentiment setups of input features were tested. Two distinct case studies were investigated. In the first case study, the evaluations were organized according to methods. The subsequent comparisons followed the grouping. In the second case study, the comparisons concerned the feature setups used as inputs. Sentiment scores were tested in the context of improving the predictive capacities of the various models used. All comparisons yielded results from an extended experimental procedure that incorporated various steps. The whole setting involved a wide range of multivariate setups, which included various sentiment time series. Multiple evaluation metrics and three different time frames were used to derive multiple-view results. Below, first, a brief presentation of related literature is given. Then, the experimental procedure is thoroughly presented, which is followed by the results. Finally, Section 5 lists the extracted conclusions.

## 2. Related Work

The continuous and ever-increasing demand for accurate forecasts across a wide range of human activity has been a key causal factor contributing to the unabated research activity occurring within the field of time series forecasting. Thus, the prediction of time series constitutes a strong pole of interest for the scientific community. Consequently, in recent decades, this interest has been reflected in a wealth of published work and important results. In this section, a brief presentation of relevant literature is given. Due to space constraints, this presentation is more indicative than exhaustive, and its purpose is just to provide a starting point for a more thorough and in-depth review.

A trivial way to distinguish the problems associated with time series forecasting would be to divide the task into two categories with respect to the type of final output. The first category includes problems where the goal is to predict whether a future value is expected to increase or decrease over a given time horizon. This task can essentially be treated as a binary classification problem. The second category includes tasks where the goal is to accurately predict the price of a time series in a specific time frame. Here, the output can take any value within a continuous interval, and hence, the prediction process can be treated as a regression problem. One can easily imagine that the difficulty of the problems belonging to the second category is greater than that of the first and that their treatment requires more complex and precise refinements. Apparently, interesting works can be found in both categories, but the context of this paper dictates a focus on the latter.

A subclass of problems regarding focus on the direction in which a time series will move features those involving the increase or decrease of closing price values of various stocks. In particular, in [1], an ensemble technique based on tree classifiers—specifically on *random forests* and *gradient boosted decision trees*—which predicts movement in various time frames is proposed. For the same purpose in [2], *support vector machines* (SVMs) are used in combination with sentiment analysis performed on data drawn from two forums considered to be the largest and most active mainstream communities in China. This paper is an attempt to predict stock price direction using SVMs and taking into account the so-called day-of-week effect. Adding sentiment variables results in up to 18% better predictions. Similar results, which indicate the superiority of SVMs compared to other classification algorithms, are also presented in [3], where well-known methods such as *linear discriminant analysis*, *quadratic discriminant analysis*, and *Elman backpropagation neural networks* are used for comparison. Encouraging results regarding the prediction of time series movement direction have also been achieved using hybrid methods, where modern schemes combining *deep neural network* architectures are applied to big data [4]—again—for the daily-based prediction of stock market prices. Regarding the second category, where the goal is to predict the specific future values of a time series and not merely its direction, the literature appears richer. This seems as if it is a fact rather expected if one takes into account the increased difficulty of the task and the high interest of the research community in pursuing the production of improved results. In the past decades, traditional statistical methods seemed to dominate the field of time series forecasting [5,6]. However, as expected, according to their general effectiveness, machine learning methods began to gain ground and dominate the field [7,8]. Traditional machine learning methods are incorporated in various time series forecasting tasks, such as using SVMs for economic data predictions [9] and short-term electric load forecasting [10], while architectures based on neural networks are also particularly popular. Regarding the latter—as this is probably the largest part of the literature regarding the use of machine learning in prediction problems—the use of such methods has covered a wide range of applications. Some indicative examples are the prediction of oil production [11] and traffic congestion [12] using deep *LSTM recurrent networks*, while an aggregated version of LSTMs has additionally been used for the short-term prediction of air pollution. Forecasting river water temperature using a hybrid model based on *wavelet-neural network* architecture was presented in [13], while *recurrent neural networks* (RNNs) have been deployed to forecast agricultural commodity prices in China [14]. Since the list of examples where neural network-based techniques show promise is long, the reader is urged to pursue additional personal research.

Furthermore, it is possibly worth mentioning the fact that in addition to increasingly sophisticated methods, techniques based on the theory of *ensembles* are also gaining ground. Roughly speaking, these are techniques in which the final result is derived through a process of using different models, with the prediction being formed from the combination of the individual ones. As an example, one can mention the ensemble scheme proposed in [15] for the prediction of energy consumption: it combines *support vector regression* (SVR), *backpropagation neural network* (BPNN), and *linear regression* (LR) learners. A similar endeavor is presented in [16], where an ensemble consisting of four learners, that is, *long short-term memory* (LSTM), *gate recurrent unit* (GRU), *autoencoder LSTM* (Auto-LSTM), and *auto-GRU*, is used for the prediction of solar energy production. A comparison involving over 300 individual and ensemble predictive layouts over Greek energy load data is presented in [17]. There, in addition to the large number of ensembles tested, the comparison also concerns both a number of forecast time frames as well as different modifications of the input data in various multivariate arrangements. In [18], an ensemble scheme based on *linear regression* (LR), *support vector regression* (SVR), and the *M5P regression tree* (M5PRT) is proposed to predict cases and deaths attributed to the COVID-19 pandemic regarding southern and central European countries.

With regard now to the context of this work, and given that its purpose—which is an extension of the work in [19]—is twofold, aiming, on the one hand, to compare a large number of methods and, on the other hand, to investigate the contribution of incorporating sentiment analysis into the forecasting process, it follows that a simple presentation of similarly targeted tasks seems quite essential. As for the first objective—that of comparing methods—there are several interesting works that have been carried out in recent years. In [20], the comparison between the traditional *ARIMA* method and *LSTMs* using economic data is investigated. A similar comparison between the two methods is implemented in [21], now aiming to predict bitcoin values, while in [22], the *gated recurrent unit* (GRU) scheme is also included in the comparison. Comparative works of the *ARIMA* method with various schemes have also been carried out, such as with *neural network auto-regressive* (NNAR) techniques [23], with the *prophet* method [24], with *LSTMs* and the *XGBOOST* method [25], as well as with *wavelet neural network* (WNN) and *support vector machines* (SVM) [26]. Although, in general, modern schemes tend to perform better than ARIMA, any absolute statement would not be representative of reality. Indeed, research focused on comprehensively reviewing the use of modern methods can provide a detailed overview of the relevant work to date. Indicatively, in [27], an extensive review of the use of artificial neural networks in time series forecasting is presented, covering studies published from 2006 onwards, over a decade. A similar survey covering the period from 2005 to 2019 and focusing on deep learning techniques with applications to financial data can be found in [28]. Furthermore, regarding the experimental evaluation of modern machine learning architectures, in [29], a thorough experimental comparison is presented, concerning seven different deep learning architectures applied to 12 different forecasting problems, using more than 50,000 time series. According to the implementation of more than 38000 models, it is argued that the architectures of *LSTMs* and *CNNs* outperform all others. In [30], the comparison of a number of methods—such as *ARIMA*, *neural basis expansion analysis* (NBEATS), and probabilistic methods based on deep learning models—applied to time series of financial data is presented. Additionally, in [31], a comparison between *CNNs*, *LSTMs*, and a hybrid model of them is given, which was deployed on data concerning the forecasting of the energy load coming from photovoltaics. There, the generated results, on the one hand, indicate the dominance of the hybrid model—emphasizing the necessity to create efficient combinatorial schemes—and, on the other, show that the models’ predictions improve by using a larger amount of data in the training set.

In relation to the second objective—which concerns the investigation of whether the use of information based on sentiment analysis regarding public opinion extracted from social networks favors the predictions—the available literature seems comparatively poorer but presents equally interesting results. The relationship between tweet board literature and financial market instruments is examined in [32], with results revealing a high correlation between stock prices and Twitter sentiments. In [33], using targeted topics to extract sentiment from social media, a model to predict stock price movement is presented. Moreover, the effectiveness of incorporating sentiment analysis into stock forecasting is demonstrated. In addition, ref. [34] is an attempt to capture the various relationships between news articles and stock trends using well-known machine learning techniques such as *random forest* and *support vector machines*. In [35], after assembling a financial-based sentiment analysis dictionary, a model incorporating the dictionary was developed and tested on data from the pharmaceutical market, exhibiting encouraging results. In [36], sentiment polarity is extracted by observing the logarithmic return of the ratio between the average stock price one minute before and one minute after the relevant stock’s news is published. Then, using *RNNs* and *LSTMs*, the direction of the stock is successfully predicted. The exploitation of sentiment analysis techniques has also been used to predict the stock market during health crises [37] such as H1N1 and, more recently, COVID-19. Possible links between social media posts and closing stock prices at specific time horizons were found. More specifically, for COVID-19, the polarity of the posts seemed to affect the stock prices after a period of about six days.

Regarding the prediction of various stock market closing prices—which is also the thematic center of this paper—in [38], data collected from Twitter are initially analyzed in terms of their sentiment scores and are then used to predict the movement of stock prices, using *naive Bayes* and *multiclass SVM* classifiers. A similar procedure was followed in [39], where *least squares support vector regression* (LSSVR) and *backpropagation neural networks* were deployed to predict the total monthly sales of vehicles in the USA, using additional sentiment information combined with historical sales data. Data collected from the online editions of international newspapers were used in [40] to predict the closing stock price values, incorporating both traditional methods, such as *ARIMA*, and newer ones, such as the Facebook *prophet* algorithm and *RNN* architectures that use as input both numerical values of the time series to be predicted as well as combinations of the polarity of extracted sentiments.

In [41], both traditional and modern machine learning methods such as *support vector machines*, *linear regression*, *naive Bayes*, and *long short-term memory* are used in combination with the incorporation of opinion data, current news, and past stock prices. In [42], sentiment analysis and *empirical model decomposition* are used so that complex time series can be broken down into simpler and easier to manage parts, together with an *attention* mechanism that attributes weight to the information considered most useful for the task being performed each time. A method based on the architecture of *LSTMs* that uses information derived from sentiment analysis together with multiple data sources is presented in [43]. Initially, textual data related to the stock in question are collected, and using methods based on *convolutional neural network* architectures, the polarity of investors’ sentiment is extracted. This information is then combined with that of the stock’s past closing prices and other technical indicators to produce the final forecast. In [44], a hybrid model that leverages deep learning architectures, such as *convolutional neural networks*, to extract and categorize investor sentiment as detected in financial forums is described. The extracted sentiments are then combined with information derived from technical financial indicators to predict future stock prices in real-world problems using *LSTM* architectures. *SVM* architectures are used on Twitter data to extract polarity in [45]. The extracted polarities are used in an incremental active learning scheme, where the continuous stream of content-changing tweets is used to predict the closing stock price of the stock market.

Sentiment analysis has also been used to predict the price of bitcoin in real time, using—and at the same time comparing—*LSTM* techniques and the classical *ARIMA* method [46], where the exploitation of the information derived from sentiment analysis has been beneficial. Similar research focused on predicting the price direction of the cryptocurrencies Bitcoin and Ethereum using sentiment analysis from data drawn from Twitter and Google Trends and given as input to a linear predictive model is presented in [47]. Interestingly, the volume of tweets affects the prediction to a greater extent than the polarity of the sentiment extracted from the tweets. Forecasting the price direction of four popular cryptocurrencies—Bitcoin, Ethereum, Ripple, and Litecoin—using machine learning techniques and data drawn from social networks is presented in [48]. Classical methods such as *neural networks* (NN), *support vector machines* (SVM), and *random forests* (RF) are compared. An interesting fact is that Twitter, roughly speaking, seems to favor the prediction of specific cryptocurrencies rather than all of them. Using sentiment analysis has also been beneficial in the field of cybersecurity. In [49], a methodology that exploits the knowledge of hacker behavior for predicting malicious events in cyberspace by performing sentiment analysis with different techniques (*VADER*, *LIWC15*, and *SentiStrength*) on data collected from hacking forums, both on the dark web and on the surface web, is presented.

The—rather diverse—list of applications in which the use of sentiment analysis techniques can improve the generated forecasts is proportional to the fields in which time series forecasting is applied since, in general, the utilization of public opinion knowledge appears to have a positive effect on the forecasting process. Some of them that have been implemented in the last five years have already been mentioned in passing, and many others can be added. Such would include predicting the course of epidemics, such as that of the Zika virus in the USA in 2016 [50] or the COVID-19 pandemic, the outcome of electoral contests [51], the prediction of the price of e-commerce products [52], and the list goes on. Given human nature and the consequent conceptual coping of the world by human subjects, sentiment analysis seems justifiably relevant in a multitude of applications. The reader is therefore encouraged to conduct additional bibliographic research.

## 3. Experimental Procedure

Information regarding the stages of the experimental procedure will now be presented. This presentation will be as detailed as possible given the necessary space constraints and content commitments in order not to disrupt the depictive nature of the paper.

It has already been mentioned that to some extent, the “core” of the present work consists of an experimental procedure that aims, in its most abstract scope, to check the efficiency, on the one hand, of a number of state-of-the-art algorithms and, on the other, of incorporating sentiment analysis into predictive schemas. Thus, a total of 16 *datasets* × 67 *combinations* × 30 *algorithms* × 3 *time-shifts* = *96,480 experiments* were conducted. The dataset consisted of time series containing the daily closing values of various stocks along with a multitude of 67 different sentiment score setups. Specifically, 16 datasets of stocks containing such closing price values were used over a three-year period, beginning on 2 January 2018 and ending on 24 December 2020. Generated sentiment scores from relevant textual data extracted from the Twitter microblogging platform were used. Three different sentiment analysis methods were deployed. The sentiment score time series and the closing values were subjected to a 7-day and a 14-day rolling mean strategy, yielding a total of 12 distinct features. Various combinations of the created features resulted in a total of 67 distinct input setups per algorithm. The calculated sentiment scores along with the closing values were then tested under both univariate and multivariate forecasting schemes. Lastly, 30 state-of-the-art methods were investigated. Below, a more thorough presentation of the aforementioned experimental setting follows.

### 3.1. Datasets

Starting with data, the process of collecting and creating the sets used will now be addressed.

#### 3.1.1. Overview

To begin with, Table 1 contains the names of the aforementioned datasets along with their corresponding abbreviations. These initial data included time series containing closing values for 16 well-known listed companies. All sets comprise three-year period data for dates ranging from 2 January 2018 to 24 December 2020.

**Figure 1:**
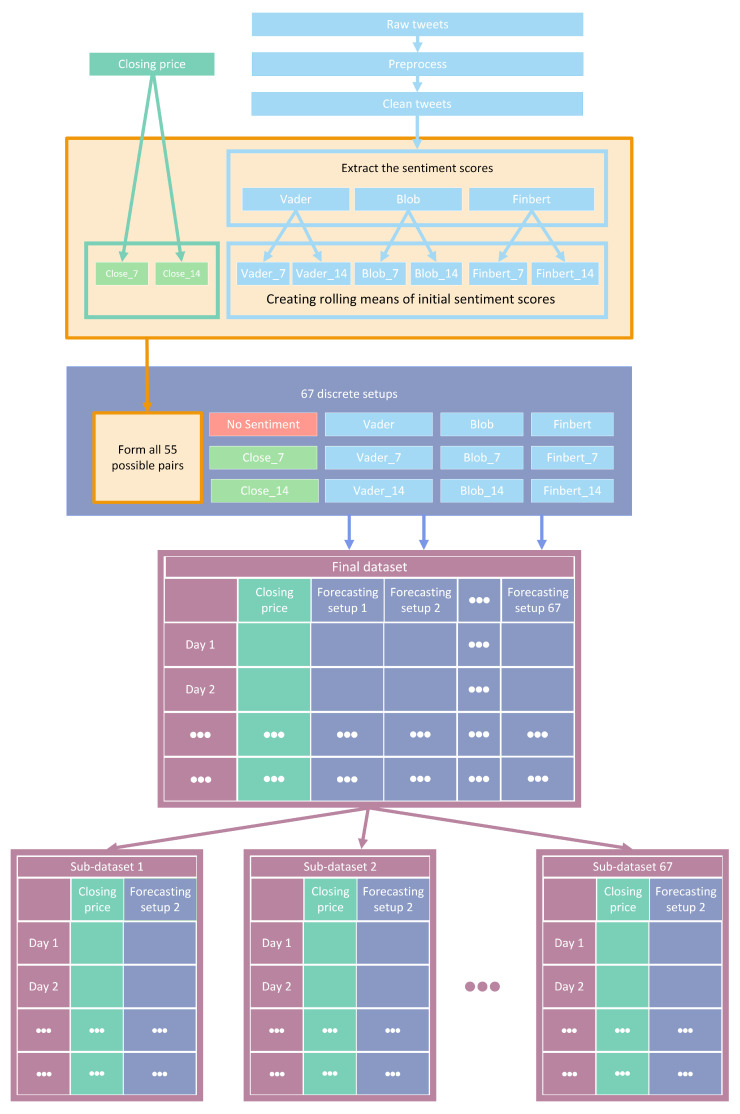
Feature setups: creation pipeline.

Essentially, the initial features were four: that is, the closing prices of each stock and three additional time series containing relative sentiment scores for the given period. Subsequently, and after applying 7- and 14-day rolling averages, a total of 14 features were extracted. Thus, for each share, the final input settings were composed by introducing altered features derived from stock values and a sentiment analysis process applied to an extended corpus of tweets. Figure 1 depicts a—rather abstractive—snapshot of the whole process from data collection to the creation of the final input setups.

#### 3.1.2. Tweets and Preprocessing

A large part of the process involved deriving sentiment scores related to stocks. Using the *Twitter Intelligence Tool* (TWINT) [53], a large number of stock-related posts written in English were downloaded from Twitter and grouped by day. TWINT is an easy-to-use yet sophisticated Python-based Twitter scraping tool. After a comprehensive search for stock-related remarks that were either directly or indirectly linked to shares under consideration, a sizable amount of text data containing daily attitudes toward stocks were created. Then, the collected textual sets underwent the various preprocessing procedures necessary in order to be passed on to the classification modules for extracting their respective sentiment scores.

**Figure 2:**
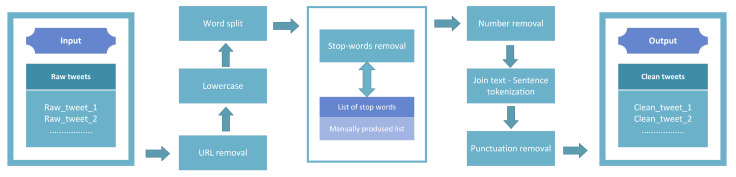
Preprocessing.

Regarding preprocessing tweets, initially, irrelevant hyperlinks and URLs were removed using the *Re* Python library [54]. Each tweet was then converted to lowercase and split into words. Then, unwanted phrases from a manually produced list and various numerical strings were also dismissed. After performing the necessary joins to restore each text to its original structure, each tweet was tokenized in terms of its sentences using the *NLTK* [55,56] library. Lastly, using the *String* [57] module, punctuation removal was applied. The whole text-preprocessing step is schematically presented in Figure 2.

#### 3.1.3. Sentiment Analysis

The subsequent process involved extracting sentiment scores from the gathered yet cleaned tweets. To perform the sentiment quantification step, three different sentiment analysis methods were utilized.

Specifically, the procedure included extracting sentiment scores from *TextBlob* [58], using the *Vader* sentiment analysis tool [59], and incorporating *FinBERT* [60]. FinBERT is a financial-based fine-tuning of the *BERT* [61] language representation model. Using each of the above methods, daily sentiment scores were extracted for each stock. The daily mean was then extracted, forming the final collection, which constituted the sentiment-valued time series of every corresponding method. Then, 7- and 14-day moving averages were applied to the previously extracted sentiment score time series. This resulted in the extraction of nine sentiment time series, which, together with the application of the aforementioned procedure to the closing price time series, led to the final number of 12 generated time series used as features. Various combinations of the above features, along with the univariate case scenario, resulted in 67 different study cases. These data constituted the distinct experimental procedures that run for every algorithm. The use of three different methods of sentiment analysis has already been mentioned. Below, a rough description of these methods is given. For further information, the reader is advised to refer to the respective papers.

- TextBlob: The *TextBlob* module is a Python-based library for performing a wide range of manipulations over text data. The specific TextBlob method used in this work is a *rule-based* sentiment-analysis scheme. That is, it works by simply applying manually created rules. This is how the value attributed to the corresponding sentiment score is calculated. An exemplified snapshot of the process would be counting the number of times a term of interest appears within a given section. This would modify the projected sentiment score values in line with the way the phrase is assessed. Here, within this experimental setup and by exploiting TextBlob’s *sentiment* property, a real number within the [−1,1] interval representing the sentiment polarity score was generated for each tweet. The algorithm’s numerical output was then averaged using the individual scores of each tweet to obtain a single sentiment value representing the users’ daily attitudes;
- Vader: *Vader* is also a straightforward *rule-based* approach for realizing general sentiment analysis. In the context of this work, the Vader sentiment analysis tool was used in order to extract a compound score produced by a normalization of sentiment values that the algorithm calculates. Specifically, given a string, the procedure outputs four values: negative, neutral, and positive sentiment values, as well as the aforementioned composite score used. A normalized average of all compound scores for each day was generated the usual way. The resulting time series contained daily sentiment scores that ranged within the [−1,1] interval;
- FinBERT: Regarding *FinBERT*, in this work, the implementation contained in [62] was utilized. Specifically, the model that was trained on *PhraseBank* presented in [63] was used. Again, first, the daily scores regarding sentiment attitudes were extracted to eventually form a daily average time series. Generally, the method is a pre-trained *natural-language-processing* (NLP) model for sentiment analysis. It is produced by simply fine-tuning the pre-trained *BERT* model over financial textual data. BERT, meaning *bidirectional encoder representations from transformers*, is an implementation of the *transformers* architecture used for natural language processing problems. The technique is basically a pre-trained representational model based on transfer learning principles. Given textual data, multi-layer deep representations are trained with a bidirectional attention strategy so that the various different contexts of each linguistic token constitute the content of the token’s embedding. Regardless of data references—here financial—the model can be fine-tuned in any domain by only using a single additional layer that addresses the specific tasks.

### 3.2. Algorithms

In this section, the methods, algorithmic schemes, and architectures employed in the experiments are listed. Additional details are given on the implementation framework and the tools used.

Regarding the algorithms used, a total of 30 different state-of-the-art methods and method variations were compared. The number of 30 methods used results from the supplementation of the set of well-known core methods with their variations. Further details can be found in the cited *tsAI* library [64], using which the implementation was carried out. However, it is this multitude of methods that apparently makes a detailed presentation practically impossible. Nevertheless, the reader is urged to track the cited papers. Table 2 contains the main algorithms utilized during the experimental procedure along with a corresponding citation. There, among others, one can notice that in addition to a multitude of state-of-the-art methods, implementations involving combinations of the individual architectures were also used. Note that in addition to the corresponding papers, information regarding the variations of the basic algorithms employed can be searched, inter alia, in notebook files taken from the library implementations.

In order to carry out the experiments, the Python library *tsAI* [64] was used. The *tsAI* module is “an open-source deep learning package built on top of Pytorch and Fastai focused on state-of-the-art techniques for time series tasks like classification, regression, forecasting” [64], and others. Here, the forecasting procedure was essentially treated as a predictive regression problem. In the experiments, the initial parameters of the respective methods from the library were preserved with the implementation environment being kept fixed for all algorithmic schemes. Thus, all algorithms compared were utilized in the most basic configuration. That way, one can gain additional insight regarding implementing high-level yet low-code programming and data analysis in real-world tasks. Of the data, 20% were used as the test set. Regarding prediction time horizons, three forecast scenarios were implemented: one single-step and two multi-step. In particular, with regard to multi-step forecasts, and leaving aside the single-step predictions, estimates were provided for a seven-day window on the one hand and a fourteen-day window on the other. The results were evaluated according to the metrics presented in the following paragraph.

**Table 2:** Algorithms.

No.	Abbreviation	Algorithm ^1^
1	FCN	Fully Convolutional Network [65]
2	FCNPlus	Fully Convolutional Network Plus [66]
3	IT	Inception Time [67]
4	ITPlus	Inception Time Plus [68]
5	MLP	Multilayer Perceptron [65]
6	RNN	Recurrent Neural Network [69]
7	LSTM	Long Short-Term Memory [70]
8	GRU	Gated Recurrent Unit [71]
9	RNNPlus	Recurrent Neural Network Plus [69]
10	LSTMPus	Long Short-Term Memory Plus [69]
11	GRUPlus	Gated Recurrent Unit Plus [69]
12	RNN_FCN	Recurrent Neural—Fully Convolutional Network [72]
13	LSTM_FCN	Long Short-Term Memory—Fully Convolutional Network [73]
14	GRU_FCN	Gated Recurrent Unit—Fully Convolutional Network [74]
15	RNN_FCNPlus	Recurrent Neural—Fully Convolutional Network Plus [75]
16	LSTM_FCNPlus	Long Short-Term Memory—Fully Convolutional Network Plus [75]
17	GRU_FCNPlus	Gated Recurrent Unit—Fully Convolutional Network Plus [75]
18	ResCNN	Residual—Convolutional Neural Network [76]
19	ResNet	Residual Network [65]
20	RestNetPlus	Residual Network Plus [77]
21	TCN	Temporal Convolutional Network [78]
22	TST	Time Series Transformer [79]
23	TSTPlus	Time Series Transformer Plus [80]
24	TSiTPlus	Time Series Vision Transformer Plus [81]
25	Transformer	Transformer Model [82]
26	XCM	Explainable Convolutional Neural Network [83]
27	XCMPlus	Explainable Convolutional Neural Network Plus [84]
28	XceptionTime	Xception Time Model [85]
29	XceptionTimePlus	Xception Time Plus [86]
30	OmniScaleCNN	Omni-Scale 1D-Convolutional Neural Network [87]

^1^ Methods and method variations used.

### 3.3. Metrics

Regarding performance evaluation, six metrics were used. The use of the different metrics serves the necessity of having not only a presentation of the conclusions of a large comparison of methods and feature and sentiment setups but also a number of diverse extractions in terms of evaluation aspects that can be used in future research. This is exactly because each of the metrics exposes the results in different aspects, and therefore, an investigation would be incomplete if it focused on just one of them. Thus, regarding evaluating results, each one of the six performance indicators utilized has advantages and disadvantages. The metrics used are:

- the *Mean Absolute Error* (MAE);
- the *Mean Absolute Percentage Error* (MAPE);
- the *Mean Squared Error* (MSE);
- the *Root Mean Squared Error* (RMSE);
- the *Root Mean Squared Logarithmic Error* (RMSLE);
- the *Coefficient of Determination* $R^{2}$.

In what follows, a rather detailed description of aspects of the aforementioned well-known evaluation metrics is given. The presentation aspires to provide details and some insight regarding the interpretation of the metrics. Below, the actual values are denoted by $y_{a_{i}}$ and the forecasts are denoted by $y_{p_{i}}$.

#### 3.3.1. MAE

First is MAE:

(1) $$MAE=\frac{1}{n}\sum_{i=1}^{n}\left|y_{p_{i}}-y_{a_{i}}\right|$$

MAE stands for the arithmetic *mean of the absolute errors*, and it is a very straightforward metric and easy to calculate. By default, in terms of the difference between the prediction and the observation, the values share the same weights. The absence of exponents in the analytic form ensures good behavior, which is displayed even when outliers are present. The target variable’s unit of measurement is the one expressing the results. MAE is a scale-dependent error metric; that is, the scale of the observation is crucial. This means that it can only be used to compare methods in scenarios where every scheme incorporates the same specific target variable rather than different ones.

#### 3.3.2. MAPE

Next is MAPE:

(2) $$MAPE=\frac{1}{n}\sum_{i=1}^{n}\frac{\left|y_{p_{i}}-y_{a_{i}}\right|}{\left|y_{a_{i}}\right|}$$

MAPE is the *mean absolute percentage error*. It is a relative and not an absolute error measure. MAPE is common when evaluating the accuracy of forecasts. It is the average of the absolute differences between the prediction and the observations divided by the absolute value of the observation. A multiplication by 100 can afterwards convert this output to a percentage. This error cannot be calculated when the actual value is zero. Instead of being a percentage, in practice, it can take values in $\left[0,\infty \right)$. Specifically, when the predictions contain values much larger than the observations, then the MAPE output can exceed 100%. Conversely, in cases where both the prediction and the observation contain low values, the output of the metric may deviate greatly from 100%. This, in turn, can lead to a misjudgment of the model’s predictive capabilities, believing them to be limited when, in fact, the errors may be low. MAPE attributes more weight to cases where the predicted value is higher than the actual one. These cases produce larger errors. Hence, using this metric is best suitable for methods with low prediction values. Lastly, MAPE, being not scale-dependent, can be used to evaluate comparisons of a variety of different time series and variables.

#### 3.3.3. MSE

The next metric is MSE:

(3) $$MSE=\frac{1}{n}\sum_{i=1}^{n}{\left(y_{p_{i}}-y_{a_{i}}\right)}^{2}$$

MSE stands for *mean squared error*. It constitutes a common forecast evaluation metric. The mean squared error is the average of the squares of the differences between the actual and predicted values. Its unit of measurement is the square of the unit of the variable of interest. Looking at the analytical form, first, the square of the differences ensures the non-negativity of the error. At the same time, it makes information about minor errors usable. It is obvious, at the same time, that larger deviations entail larger penalties, i.e., a higher MSE. Thus, outliers have a big influence on the output of the error; that is, the existence of such extreme values has a significant impact on the measurements and, consequently, the evaluation. Furthermore, and in a sense the other way around, when differences are less than 1, there is a risk of overestimating the predictive capabilities of the model. Given the error’s differentiability, as one can observe, it can easily be optimized.

#### 3.3.4. RMSE

Moving on to RMSE:

(4) $$RMSE=\sqrt{\frac{1}{n}\sum_{i=1}^{n}{\left(y_{p_{i}}-y_{a_{i}}\right)}^{2}}$$

RMSE stands for *root mean squared error*. It is a common metric for evaluating differences between estimated values and observations. To compute it, apparently, one just calculates the root of the mean squared error. From the numerical formulation, one can think of the metric as an abstraction that captures the representation of something of an average distance between the actual values and the predictions. That is, if one ignores the denominator, then one can observe the formula as being the Euclidean distance. The subsequent interpretation of the metric as a kind of normalized distance comes out of the act of division by the number of observations. Here also, the existence of outliers has a significant impact on the output. In terms of interpreting error values, the RMSE is expressed in the same units as the target variable and not in its square, as in the MSE, making its use straightforward. Finally, the metric is scale-dependent; hence, one can only use it to evaluate various models or model variations given a particular fixed variable.

#### 3.3.5. RMSLE

The next metric is also an error. The formula for RMSLE is as follows:

(5) $$RMSLE=\sqrt{\frac{1}{n}\sum_{i=1}^{n}{\left(\log\left(y_{p_{i}}+1\right)-\log\left(y_{a_{i}}+1\right)\right)}^{2}}$$

RMSLE stands for Root Mean Squared Logarithmic Error. The RMSLE metric seems as if it is a modified version of the MSE. Using this modification is preferred when predictions display significant deviations. RMSLE uses logarithms of both the observations and predicted values while ensuring non-zero values in the logarithms through the appropriate simple unit additions appearing in the formula. This modified version is resistant to the existence of outliers and noise, and it smooths the penalty that the MSE imposes in cases in which predictions deviate significantly from observations. The metric cannot be used when there are negative values. RMSLE can be interpreted as a relative error between observations and forecasts. This can be made evident by simply applying the following property to the radicand term of the square root:

(6) $$\log\left(y_{p_{i}}+1\right)-\log\left(y_{a_{i}}+1\right)=\log\left(\frac{y_{p_{i}}+1}{y_{a_{i}}+1}\right)$$

Since RMSLE gives more weight to cases where the predicted value is lower than the actual value, it is quite a useful metric for types of predictions where similar conditions require special care for the reliability of the application in real-world conditions, where lower forecasts may lead to specific problems.

#### 3.3.6. R${}^{2}$

The last metric is the coefficient of determination $R^{2}$:

(7) $$R^{2}=1-\frac{SS_{RES}}{SS_{TOT}}=1-\frac{\sum_{i=1}^{n}{\left(y_{p_{i}}-y_{a_{i}}\right)}^{2}}{\sum_{i=1}^{n}{\left(y_{p_{i}}-\bar{y}\right)}^{2}}$$

The coefficient of determination $R^{2}$ is not an error evaluation metric. It is the ratio depicted in the above equation. This metric is essentially not a measure of model reliability. $R^{2}$ is a measure of how good a fit is: a quantification of how well a model *fits* the data. Its values typically range from 0 to 1. A rather simple interpretation would be this: the closer to 1 the value of the metric is, the better the model fits the observations, i.e., the predictions are closer, in terms of their values, to the observations. Thus, the value 0 corresponds to cases where the explanatory variables do not explain the variance of the dependent variable at all. Conversely, the value 1 corresponds to cases where the explanatory variables fully explain the dependent variable. However, this interval does not strictly constitute the set of values of the metric. There are conditions in which $R^{2}$ could take negative values. Observing the formula, one can identify the above as permissible. In such cases, the model performs worse in fitting the data than a simple horizontal line, essentially being unable to follow the trend. Lastly, values outside the above range indicate either an inadequate model or other flaws in its implementation.

## 4. Results

Returning to the dual objective of this work, the two case studies whose results will be presented in this chapter were:

- On the one hand, the comparison of a large number of time series forecasting contemporary algorithms;
- On the other hand, the investigation of whether knowledge of public opinion, as reflected in social networks and quantified using three different sentiment analysis methods, can improve the derived predictions.

Accordingly, the presentation of the results of the experimental process is split into two distinct parts. In what follows, both various statistical analysis and visualization methods are incorporated. However, it should be noted that the number of comparisons performed yielded a quite large volume of results. Specifically, as already pointed out, in each case, the performance of the 30 predictive schemes and the 67 different feature setups was investigated over three different time frames (1, 7, and 14 day shifts). Note that these three time-shifting options have no—or at least no intended—financial consequences. Here, the primary goal in designing the framework was to forecast the stock market over short time frames, such as a few days. Then, an expansion was made to investigate the performance of both methods and feature setups over longer periods of time. Each of these schemas was evaluated with six different metrics, while the process was repeated for each of the datasets. Consequently, it becomes clear that the complete tables with the numerical results cannot contribute satisfactorily to the understanding of the conclusions drawn. Below, following a necessary brief reminder of the process, results are presented.

As has already been mentioned, during the procedure, for each of the stocks, the following strategy was followed: each of the thirty algorithms to be compared was “ran” 67 times, each time accepting as input one of the different feature setups. This was repeated three times, once for each of the three forecast time frames. In each of the above runs, the six metrics used in the evaluation of the results were calculated. The comparison of the algorithms was performed by using Friedman’s statistical tests in terms of feature setups for each of the time shifts. Thus, given setups and stocks, the ranking of the methods per evaluation metric was extracted according to the use of the Friedman test [88]. Therefore, regarding this case study, a total of 67 × 6 × 3 = 1206 *statistical tests* were executed. In a similar way, the Friedman rankings of input feature setups were estimated in terms of metrics and time shifts, given the various algorithms and stocks. Here, a total of 30 × 6 × 3 = 540 *statistical tests* were performed. An additional abstraction of the results was derived as follows: For each of the 30 methods, the average rank achieved by each method in terms of feature setups and shares was calculated. So, for each metric and each of the three time frames, a more comprehensive display of the information was obtained based on the average value of the different setups. In an identical way, in the case of checking the effectiveness of features, the average value of the 30 algorithms for each of the 67 different input setups was calculated in each case. In both cases, the ranking was calculated based on the positions produced by the Friedman test, while at the same time, with the Nemenyi post hoc test [89] that followed, every schema was checked pair-wise for significant differences. The results of the Nemenyi post hoc tests are shown in the corresponding Critical Difference diagrams (CD-diagrams), in which methods that are not significantly different are joined by black horizontal lines. Two methods are considered not significantly different when the difference between their mean ranks is less than the CD value.

Next, organized in both cases based on time frames, the results concerning the comparison of the forecast algorithms are presented, which are followed by those regarding the feature setups.

### 4.1. Method Comparison

The presentation begins with results concerning the investigation of methods. The results are presented per forecast time shift. In each case, the Friedman Ranking results for all six metrics are listed. To save space, only methods that occupy the top ten positions of the ranking are listed. Full tables are available at: shorturl.at/FTU06 (accessed on 15 January 2023). The CD diagrams follow. There, we can visually observe which of the methods exhibit similar behavior and which differ significantly. Finally, box plots of results per metric are presented, again for the best 10 methods. The box plots present in a graphical and concise manner information concerning the distribution of the aforementioned data, that is, in our case, the average values of the sentiment setups per algorithm for all stocks. In particular, one can derive information about the maximum and minimum value of the data, the median, as well as the 1st and 3rd quartile values isolated by 25% and 75% of the observations, respectively.

#### 4.1.1. Time Shift 1

With respect to the one-day forecasts, Table A1 lists the Friedman Ranking results for the top 10 scoring methods per metric. Although there is no single method that dominates all metrics and significant reorderings are also observed in the table positions, the TCN method achieves the best ranking in three out of six metrics (MAPE, R2, and RMSLE) and is always in the top four. Furthermore, from the box plots, it is evident that TCN has by far the smallest range of values.

Apart from this, in all metrics, GRU_FCN is always in the top five. It is also observed that LSTM_FCN and LSTMPlus behave equally well. The latter shows a drop in the MAPE metric, but in all other cases, it is in the top three, while in two metricsm it ranks first. It should also be noted that the LSTMPlus method ranks first in two metrics, namely MAE and RMSE. In terms of R${}^{2}$ and RMSLE, it occupies the second position of the ranking, while regarding MSE, LSTMPlus ranks third. However, at the same time, according to MAPE, the method is not even in the top ten. Thus, as will be seen in the following, TCN is the consistent choice.

**Figure 3:**
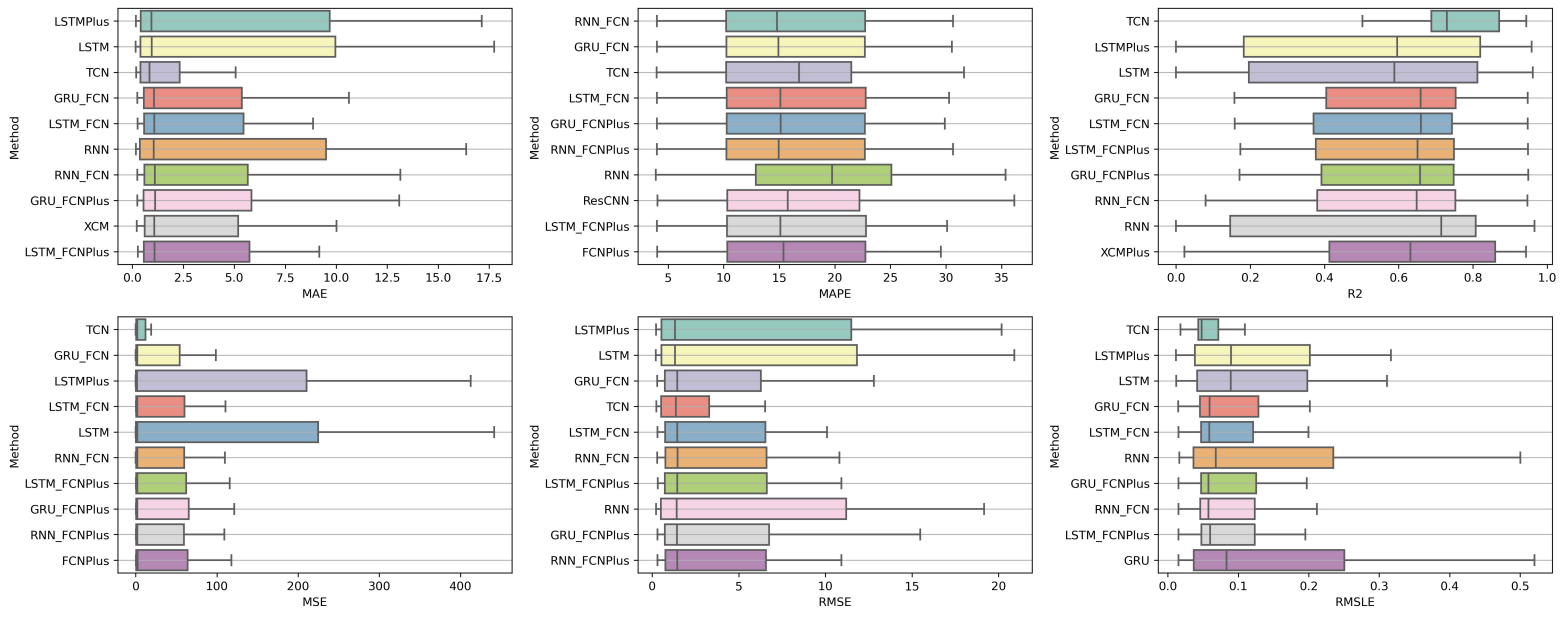
Box Plots: Methods—Shift 1.

**Figure 4:**
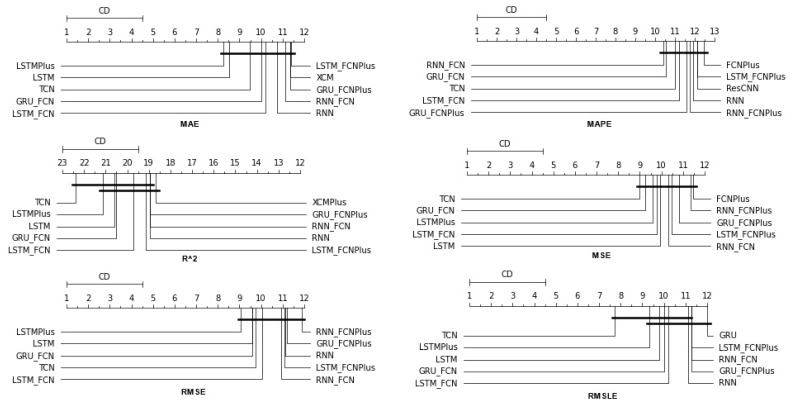
CD Diagrams: Methods—Shift 1.

The results produced by Friedman’s statistical test, in terms of the six metrics, are presented in Table A1, while the corresponding CD diagrams and box plots are depicted in Figure 3 and Figure 4.

#### 4.1.2. Time Shift 7

**Figure 5:**
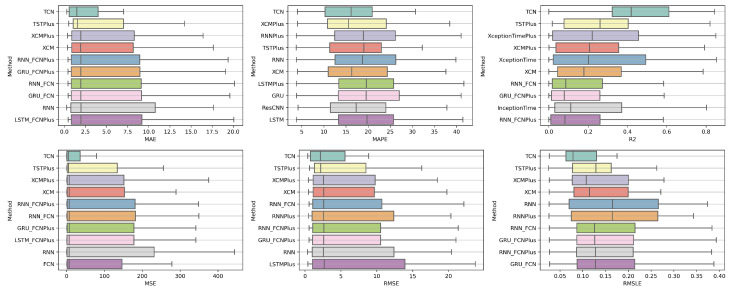
Box Plots: Methods—Shift 7.

**Figure 6:**
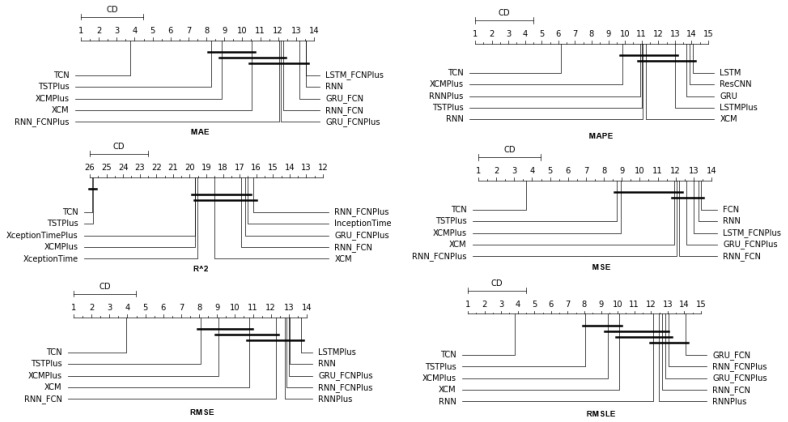
CD Diagrams: Methods—Shift 7.

At the one-week forecast time frame, the algorithms that occupy the top positions in the ranking produced by the statistical control appear to have stabilized. The corresponding ranking produced by the Friedman statistical test regarding the ten best methods with respect to the six metrics is presented in Table A2. In all metrics, the TCN method ranks first. From the CD diagrams, it can be seen that in all metrics—except for R2—this superiority is also validated by the fact that this method differs significantly from the others. Box plots show the method also having the smallest range around the median. Figure 5 and Figure 6 contain the relevant results in the form of box plots and CD-diagrams.

Other methods that clearly show some dominance over the rest in terms of given performance ratings are, on the one hand, TSTPlus, which ranks second in all metrics except MAPE, and, on the other hand, XCMPlus and XCM, which are mostly found in the top five. In general, the same methods can be found in similar positions in all metrics, with minor rank variations. In addition, the statistical correlations between the methods are shown in the CD diagram plots.

#### 4.1.3. Time Shift 14

In the forecast results with a two-week shift, a relative agreement can be seen in the top-ranking algorithms with those of the one-week frames. The ranking produced by the Friedman statistical test for the ten best methods with respect to the six metrics is presented in Table A3.

**Figure 7:**
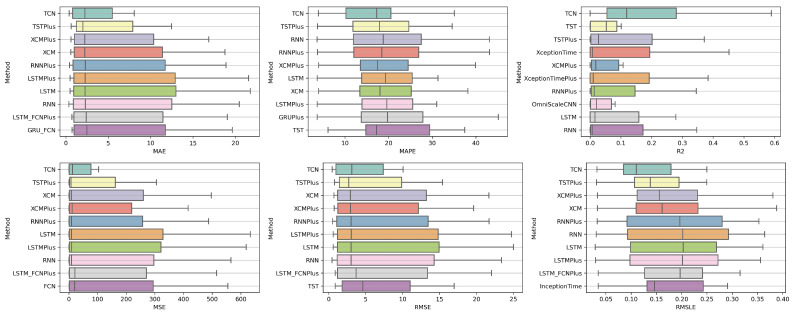
Box Plots: Methods—Shift 14.

**Figure 8:**
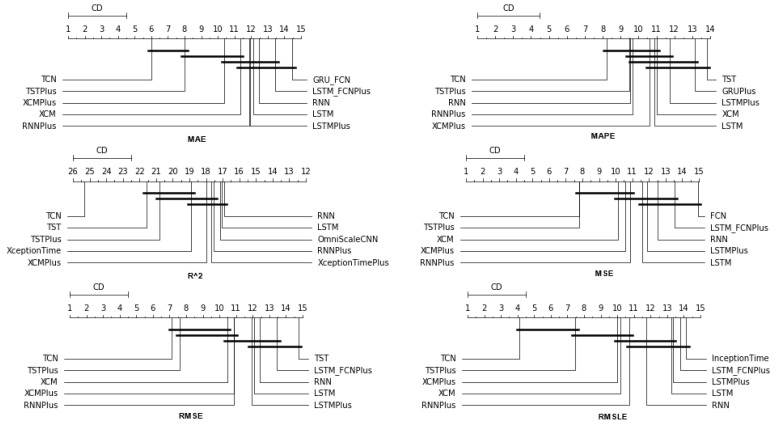
CD Diagrams: Methods—Shift 14.

Once more, TCN ranks first in all metrics. TSTPlus again ranks second in all metrics except for R2, where it ranks third. In almost all cases, XCMPlus and RNNPlus appear in the top five. Likewise, as in the previous time shift, there is a relative agreement in the methods appearing in the corresponding positions regarding all metrics. Moreover, according to the above, an argument regarding the general superiority of the TCN method in this particular scenario is easily obtained. An obvious predominance of the TCN method is established. The corresponding CD diagrams and box plots for the 10 best performing algorithms are seen in Figure 7 and Figure 8.

### 4.2. Feature Setup Comparison

Now, we are moving on to the findings of the second case study, which concern, on the one hand, the investigation of whether the use of sentiment analysis contributes to the improvement of the extracted predictions and, on the other hand, the identification of specific feature setups whose use improves the model’s predictive ability.

Again, the results of the experimental procedure will be presented separately for the three forecast time frames. Likewise, due to the volume of results, only the 10 most promising feature setups will be listed. These were again derived based on the Friedman classification of the averages calculated for each of them, taking into account the predictions in the use of the 30 forecast methods used. The full rankings of all 67 setups can be found at shorturl.at/alqwx (accessed on 13 December 2022). For the presentation below, again, the corresponding CD diagrams and box plots were used.

#### 4.2.1. Time Shift 1

**Figure 9:**
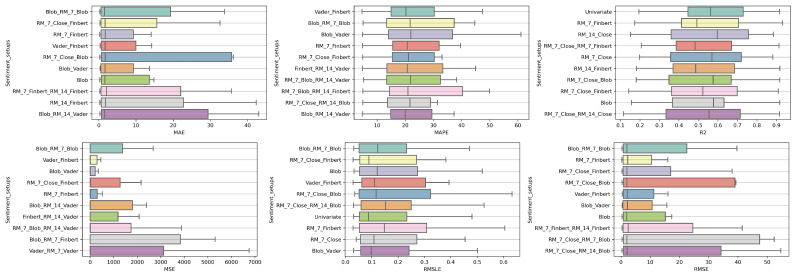
Box Plots: Features—Shift 1.

**Figure 10:**
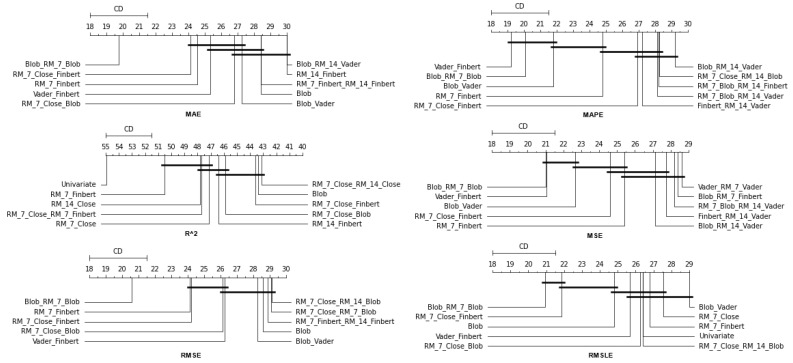
CD Diagrams: Features—Shift 1.

Starting with the results concerning one-day depth forecasting, one notices that the univariate version, in which the forecasts are based only on the stock price of the previous days, ranks first only in the case of the R${}^{2}$ metric. In fact, in three metrics, the univariate version is not even in the top twenty of the ranking (See Figure 9 and Figure 10).

Another interesting observation would be that even though there are rerankings of the sentiment setups in terms of their performance on the six metrics, the Blob_RM_7_Blob setup—that is, the setup incorporating Blob and Rolling Mean 7 Blob along with the closing values time series—although it does not score well in the ranking regarding R${}^{2}$, it is, on the one hand, at the top ranking in four metrics, that is, MAE, MSE, RMSE, RMSLE, and, on the other hand, second in MAPE. Moreover, from the results, it becomes evident that an argument in favor of using sentiment analysis in multivariate time series layouts, even in the case where the forecasts concern one-day depth, is, at least, relevant. At the same time, using smoothed versions of both the sentiment time series and those containing the closing stock price values appears to be beneficial in general.

#### 4.2.2. Time Shift 7

**Figure 11:**
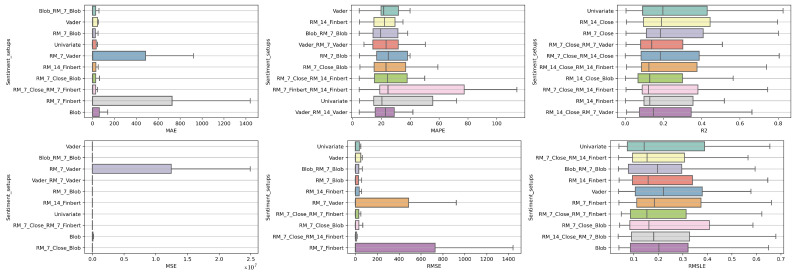
Box Plots: Features—Shift 7.

**Figure 12:**
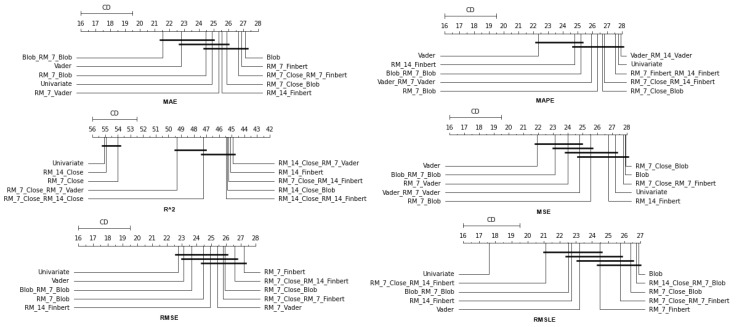
CD Diagrams: Features—Shift 7.

Regarding the time frame of one week, one can notice that the use of the univariate version is marginally ranked first in three metrics, namely, the R${}^{2}$, RMSE and RMSLE, while in two metrics, the Vader sentiment setup appears to be superior, actually being, at the same time, in second place regarding the MAPE and RMSE metrics and fifth regarding the RMSLE (Figure 11 and Figure 12).

It is also notable that Blob_RM_7_Blob, which appeared to perform particularly well during the one-day shift, remains in the top three rankings in five of the six metrics. More generally, once again, one notices that there are rearrangements, especially in the central positions of the table. However, given the small differences in performance between the different setups, this should not be considered unreasonable. Overall, the picture still points in favor of using multivariate inputs containing sentiment data.

#### 4.2.3. Time Shift 14

**Figure 13:**
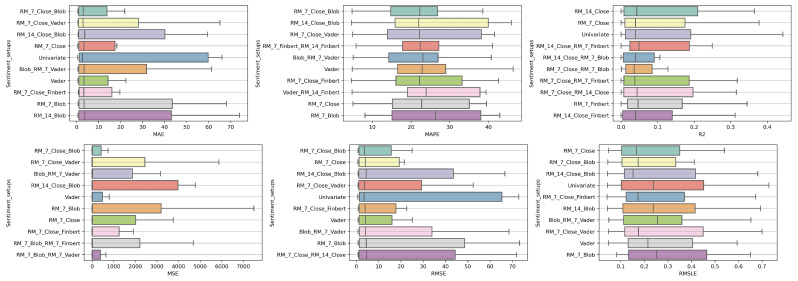
Box Plots: Features—Shift 14.

**Figure 14:**
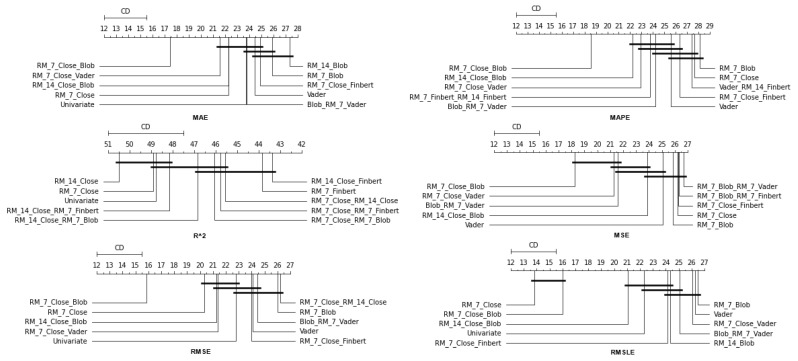
CD-Diagrams: Features—Shift 14.

Finally, regarding the two-week time frame, a first observation is that in relation to the R${}^{2}$, a feature setup that does not contain sentiment data dominates. This pattern is also present in the previous time shifts (See Figure 13 and Figure 14).

In addition, although there are metrics in which the univariate version is in the top ten, in these cases, the difference in its performance with those in the first positions is quite significant. This is easily seen from the CD diagrams: there are no connections with setups that appear in the top positions. At the seven-day time lag, it was observed that the univariate version prevailed in three cases. However, as one examines the 14-day time shift, one notices that the superiority of methods that use sentiment data is reinforced.

At the same time, combinations containing the closing price appear in the first positions of the table more often than in the previous two setups. Furthermore, it is observed that the setup that dominates four of the six metrics is RM_7_Close_Blob. These metrics are MAE, MAPE, MSE, and RMSE. The RM_7_Close_Blob feature setup is the one that incorporates both a smoothed version of the closing values as well as sentiment scores. Thus, the use of weighted averages in the original time series along with the incorporation of sentiment scores is mostly shown to be optimal regardless of the individual choice of a specific layout. Methodologically, the utilization of both has an improving effect.

## 5. Conclusions

Some general conclusions drawn from the whole experimental procedure will now be addressed. The discussion will follow the binary separation of the preceding case studies.

### 5.1. Methods

The first case study of the paper consisted of a comparison of 30 methods for time series forecasting. Within the above-discussed experimental context, the extracted results are such as to safely allow a conclusion regarding the superiority of the TCN method over the rest. This is the case because, in the vast majority of comparisons, it excels, being, for the most part, at the top of the Friedman ranking. In particular, the only cases where it does not outperform all the rest are found in the single-day time frame predictions. In fact, from the CD diagrams, one can extract the additional fact that in many cases, the superiority of the aforementioned method is marked by a significant difference. Furthermore, in addition to the TCN method, other methods whose predictive capacities can be considered significant were identified. TSTPlus is one of them, as it produces significant results, particularly over longer time horizons. XCMPlus is another.

**Figure 15:**
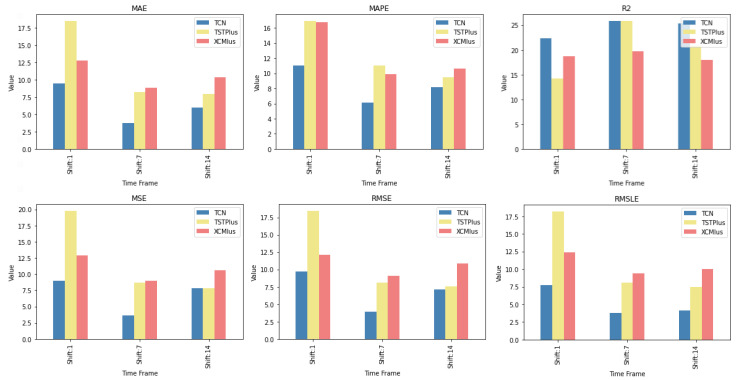
TCN, TSTPlus and XCMPlus relative rankings.

In Figure 15, one can see the relative rankings of these three methods per time shift. The values in Figure 15 correspond to the values of Table A1, Table A2 and Table A3. Regarding the one-day forecast window, LSTMPlus is an additional option, as is the combination of GRU and FCN. However, an additional point to note here is that the individual method differences are less clear in their significance. On the contrary, there can also be conclusions regarding methods whose behavior was not evaluated, on average, as satisfactory. In particular, specific methods that are always ranked last in all scenarios were identified. Specifically, TSiTPlus ranks last in all three scenarios across all metrics. In addition to this, there are methods, such as Transformer Model, XceptionTime, and XceptionTimePlus, which are always at the bottom of the table in the vast majority of cases. In conclusion, given the limitations and further prerequisites developed throughout this paper, TCN can be easily recommended.

### 5.2. Feature and Sentiment Setups

In relation to the second case study, the consideration of the results also points in some important directions. Of these, the main conclusion drawn seems to be that the use of information derived from both smoothed versions of the initial time series and sentiment analysis shows, in most cases, to have a beneficial effect on the derived forecasts. Not using sentiments in the feature setup of the inputs dominates the rest only in a small number of cases, and, as confirmed by the CD diagrams, only in two of them is this difference significant.

Moreover, the answer to whether the use of sentiment setups specifically leads to the extraction of more accurate forecasts, as evidenced by the individual layouts of the weighted results, seems to be that, in general, sentiment analysis improves forecasts. Of course, it is also reasonable to investigate whether there is a specific sentiment setup that outperforms the rest. This would also lead to an assessment of the performance of the three sentiment analysis methods used. However, the answer to this question needs further investigation. However, even with the possibility of further inquiries within the framework of the experimental setup presented here, it is still not certain that firm conclusions will be drawn. Here, while such setups can be found for each time horizon, there is not one that dominates all three.

In order, however, to illustrate a relative ranking of the three sentiment analysis methodologies used, regardless of the particular variation involved, an additional table was created. All variations of each method were placed under a corresponding class. The Friedman-aligned ranks [90] were then calculated. Hence, in order to draw a clearer picture of the way the three employed approaches to sentiment analysis performed, three sentiment classes were formed, one matching each of the previously described sentiment analysis methods. The arithmetic mean of all the sentiment setups that solely contain different variations of a particular sentiment analysis algorithm, that is, only one of the three incorporated, is used to represent the corresponding class concerning each metric. In other words, each class represents a sentiment analysis method, and each class corresponds to six sentiment setups that contain variations exclusively of the technique in question. Specifically, a representative value of a class, as it pertains to a particular *method*, is formed by the following setups: *method*, RM7*method*, RM14*method*, *method* + RM7*method*, *method* + RM14*method*, and RM7*method* + RM14*method*. The sum is then divided by six, which is apparently the number of setups, and this result is the output value to be depicted. This way, setups produced either by combining the various sentiment analysis methods or by using the target variable in variants containing rolling means are excluded in order to compare only the relative performances of the three individual techniques and their variations.

**Figure 16:**
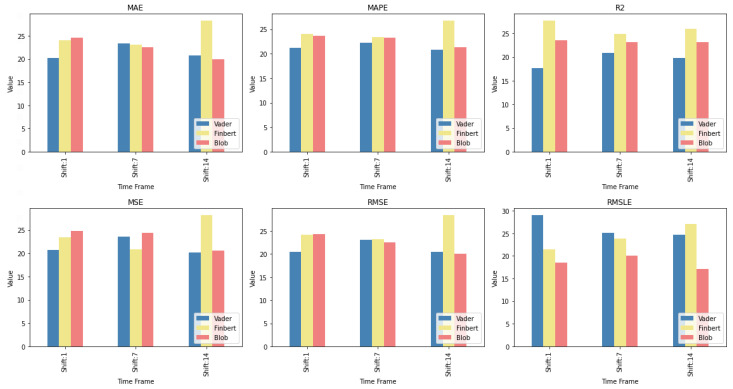
Sentiment rankings.

Figure 16 illustrates these relative rankings of the three sentiment analysis methods per time shift. One can observe the relative performances in terms of individual wins with respect to each metric and time shift: the *Blob* and *Vader* classes top the ranking seven times each, while the *Finbert* class only has four wins. Again, a conclusion in terms of an obvious generality regarding a specific algorithm does not appear. Nevertheless, the identification of groups of such setups, even at the level of a specific time frame, can be particularly useful, with the methodology for the selection of individual setups needing more investigation.

## Figures and Tables

**Table 1:** Stock datasets.

No	Dataset	Stocks
1	AAL	American Airlines Group
2	AMD	Advanced Micro Devices
3	AUY	Yamana Gold Inc.
4	BABA	Alibaba Group
5	BAC	Bank of America Corporation
6	ET	Energy Transfer L.P.
7	FCEL	FuelCell Energy Inc.
8	GE	General Electric
9	GM	General Motors
10	INTC	Intel Corporation
11	MRO	Marathon Oil Corporation
12	MSFT	Microsoft Corporation
13	OXY	Occidental Petroleum Corporation
14	RYCEY	Rolls-Royce Holdings
15	SQ	Square
16	VZ	Verizon Communications

## Data Availability

URLs of the full Friedman Ranking results. (i) Methods rankings: shorturl.at/FTU06 (accessed on 15 January 2023). (ii) Feature setup rankings: shorturl.at/alqwx (accessed on 15 January 2023).

## Appendix A

**Table A1:** Friedman results: Algorithms—Shift 1.

	MAE		MAPE		R${}^{2}$	
	Method	Friedman Score	Method	Friedman Score	Method	Friedman Score
**1st**	LSTMPlus	8.266667	RNN_FCN	10.4	TCN	22.4
**2nd**	LSTM	8.533333	GRU_FCN	10.53333	LSTMPlus	21.13333
**3rd**	TCN	9.466667	TCN	11	LSTM	20.6
**4th**	GRU_FCN	10	LSTM_FCN	11.2	GRU_FCN	20.53333
**5th**	LSTM_FCN	10.2	GRU_FCNPlus	11.6	LSTM_FCN	19.73333
**6th**	RNN	10.73333	RNN_FCNPlus	11.73333	LSTM_FCNPlus	19.13333
**7th**	RNN_FCN	11.13333	RNN	11.93333	GRU_FCNPlus	18.93333
**8th**	GRU_FCNPlus	11.33333	ResCNN	12.13333	RNN_FCN	18.93333
**9th**	XCM	11.33333	LSTM_FCNPlus	12.13333	RNN	18.93333
**10th**	LSTM_FCNPlus	11.4	FCNPlus	12.46667	XCMPlus	18.66667
	**MSE**		**RMSE**		**RMSLE**	
	**Method**	**Friedman Score**	**Method**	**Friedman Score**	**Method**	**Friedman Score**
**1st**	TCN	9	LSTMPlus	9.066667	TCN	7.733333
**2nd**	GRU_FCN	9.266667	LSTM	9.6	LSTMPlus	9.333333
**3rd**	LSTMPlus	9.6	GRU_FCN	9.6	LSTM	9.8
**4th**	LSTM_FCN	9.8	TCN	9.733333	GRU_FCN	10
**5th**	LSTM	9.933333	LSTM_FCN	10.06667	LSTM_FCN	10.2
**6th**	RNN_FCN	10.33333	RNN_FCN	10.93333	RNN	11.13333
**7th**	LSTM_FCNPlus	10.46667	LSTM_FCNPlus	11.06667	GRU_FCNPlus	11.26667
**8th**	GRU_FCNPlus	10.8	RNN	11.13333	RNN_FCN	11.26667
**9th**	RNN_FCNPlus	11.33333	GRU_FCNPlus	11.2	LSTM_FCNPlus	11.26667
**10th**	FCNPlus	11.46667	RNN_FCNPlus	11.86667	GRU	12

**Table A2:** Friedman results: Algorithms—Shift 7.

	MAE		MAPE		R${}^{2}$	
	Method	Friedman Score	Method	Friedman Score	Method	Friedman Score
**1st**	TCN	3.733333	TCN	6.133333	TCN	25.86667
**2nd**	TSTPlus	8.266667	XCMPlus	9.866667	TSTPlus	25.8
**3rd**	XCMPlus	8.866667	RNNPlus	10.93333	XceptionTimePlus	19.7
**4th**	XCM	10.53333	TSTPlus	11	XCMPlus	19.66667
**5th**	RNN_FCNPlus	12.06667	RNN	11.06667	XceptionTime	19.53333
**6th**	GRU_FCNPlus	12.13333	XCM	11.26667	XCM	18.53333
**7th**	RNN_FCN	12.26667	LSTMPlus	13	RNN_FCN	16.9
**8th**	GRU_FCN	13.2	GRU	13.66667	GRU_FCNPlus	16.66667
**9th**	RNN	13.53333	ResCNN	13.86667	InceptionTime	16.5
**10th**	LSTM_FCNPlus	13.53333	LSTM	14.06667	RNN_FCNPlus	16.16667
	**MSE**		**RMSE**		**RMSLE**	
	**Method**	**Friedman Score**	**Method**	**Friedman Score**	**Method**	**Friedman Score**
**1st**	TCN	3.666666667	TCN	3.933333333	TCN	3.8
**2nd**	TSTPlus	8.733333333	TSTPlus	8.066666667	TSTPlus	8.066666667
**3rd**	XCMPlus	8.933333333	XCMPlus	9.066666667	XCMPlus	9.4
**4th**	XCM	11.93333333	XCM	10.8	XCM	10.06666667
**5th**	RNN_FCNPlus	12.06666667	RNN_FCN	12.26666667	RNN	12.13333333
**6th**	RNN_FCN	12.2	RNNPlus	12.8	RNNPlus	12.46666667
**7th**	GRU_FCNPlus	12.6	RNN_FCNPlus	12.86666667	RNN_FCN	12.66666667
**8th**	LSTM_FCNPlus	13	GRU_FCNPlus	13	GRU_FCNPlus	12.86666667
**9th**	RNN	13.26666667	RNN	13.06666667	RNN_FCNPlus	13.06666667
**10th**	FCN	13.4	LSTMPlus	13.66666667	GRU_FCN	14.06666667

**Table A3:** TFriedman results: Algorithms—Shift 14.

	MAE		MAPE		R${}^{2}$	
	Method	Friedman Score	Method	Friedman Score	Method	Friedman Score
**1st**	TCN	6	TCN	8.2	TCN	25.33333333
**2nd**	TSTPlus	8	TSTPlus	9.466666667	TST	21.6
**3rd**	XCMPlus	10.4	RNN	9.533333333	TSTPlus	20.8
**4th**	XCM	11.33333333	RNNPlus	9.666666667	XceptionTime	18.9
**5th**	RNNPlus	11.86666667	XCMPlus	10.6	XCMPlus	17.96666667
**6th**	LSTMPlus	11.93333333	LSTM	10.86666667	XceptionTimePlus	17.7
**7th**	LSTM	12.13333333	XCM	11	RNNPlus	17.53333333
**8th**	RNN	12.46666667	LSTMPlus	11.73333333	OmniScaleCNN	17.13333333
**9th**	LSTM_FCNPlus	13.46666667	GRUPlus	13.13333333	LSTM	17.03333333
**10th**	GRU_FCN	14.46666667	TST	13.8	RNN	16.93333333
	**MSE**		**RMSE**		**RMSLE**	
	**Method**	**Friedman Score**	**Method**	**Friedman Score**	**Method**	**Friedman Score**
**1st**	TCN	7.8	TCN	7.133333333	TCN	4.133333333
**2nd**	TSTPlus	7.8	TSTPlus	7.6	TSTPlus	7.466666667
**3rd**	XCM	10.13333333	XCM	10.46666667	XCMPlus	10
**4th**	XCMPlus	10.6	XCMPlus	10.86666667	XCM	10.2
**5th**	RNNPlus	10.86666667	RNNPlus	10.86666667	RNNPlus	10.73333333
**6th**	LSTM	11.6	LSTMPlus	11.93333333	RNN	11.73333333
**7th**	LSTMPlus	11.86666667	LSTM	12.06666667	LSTM	13.26666667
**8th**	RNN	12.53333333	RNN	12.4	LSTMPlus	13.33333333
**9th**	LSTM_FCNPlus	13.53333333	LSTM_FCNPlus	13.46666667	LSTM_FCNPlus	13.8
**10th**	FCN	14.93333333	TST	14.73333333	InceptionTime	14.13333333

## Appendix B

### Appendix B.1

Please use the abbreviation table below to read the corresponding results of the Friedman Ranks.

**Table A4:** Feature Setups and Abbreviations.

No.	Abbreviation	Feature Setup
1	U	Univariate
2	B	Blob
3	V	Vader
4	F	Finbert
5	RM7C	Rolling Mean 7 Closing Value
6	RM14C	Rolling Mean 14 Closing Value
7	RM7B	Rolling Mean 7 Blob
8	RM14B	Rolling Mean 14 Blob
9	RM7V	Rolling Mean 7 Vader
10	RM14V	Rolling Mean 14 Vader
11	RM7F	Rolling Mean 7 Finbert
12	RM14F	Rolling Mean 14 Finbert

### Appendix B.2

**Table A5:** Friedman results: feature setups—Shift 1.

	MAE		MAPE		R${}^{2}$	
	Feature Setup	Friedman Score	Feature Setup	Friedman Score	Feature Setup	Friedman Score
**1st**	B_RM7B	19.73333	V_F	19.2	U	54.93333
**2nd**	RM7C_F	24.13333	B_RM7B	20.06667	RM7F	50.53333
**3rd**	RM7F	24.53333	B_V	21.8	RM14C	47.8
**4th**	V_F	25.33333	RM7F	24.8	RM7C_RM7F	47.73333
**5th**	RM7C_B	26.8	RM7C_F	26.93333	RM7C	47.13333
**6th**	B_V	27.26667	F_RM14V	27.2	RM14F	46.4
**7th**	B	28.4	RM7B_RM14V	28.13333	RM7C_B	45.86667
**8th**	RM7F_RM14F	28.4	RM7B_RM14F	28.2	RM7C_F	43.6
**9th**	RM14F	30	RM7C_RM14B	28.26667	B	43.4
**10th**	B_RM14V	30	B_RM14V	29.2	RM7C_RM14C	43.13333
	**MSE**		**RMSE**		**RMSLE**	
	**Feature Setup**	**Friedman Score**	**Feature Setup**	**Friedman Score**	**Feature Setup**	**Friedman Score**
**1st**	B_RM7B	21	B_RM7B	20.6	B_RM7B	20.93333
**2nd**	V_F	21.06667	RM7F	24.13333	RM7C_F	21.86667
**3rd**	B_V	22.66667	RM7C_F	24.2	B	24.8
**4th**	RM7C_F	24.6	RM7C_B	26.13333	V_F	25.66667
**5th**	RM7F	25.4	V_F	26.26667	RM7C_B	26.26667
**6th**	B_RM14V	27.13333	B_V	28.26667	RM7C_RM14B	26.4
**7th**	F_RM14V	27.73333	B	28.6	U	26.4
**8th**	RM7B_RM14V	28.2	RM7F_RM14F	28.86667	RM7F	26.8
**9th**	B_RM7F	28.4	RM7C_RM7B	29.06667	RM7C	27.53333
**10th**	V_RM7V	28.6	RM7C_RM14B	29.13333	B_V	29

**Table A6:** Friedman results: feature setups—Shift 7.

	MAE		MAPE		R${}^{2}$	
	Feature Setup	Friedman Score	Feature Setup	Friedman Score	Feature Setup	Friedman Score
**1st**	B_RM7B	21.53333	V	22.33333	U	55.06667
**2nd**	V	22.8	RM14F	24.8	RM14C	54.93333
**3rd**	RM7B	24.46667	B_RM7B	25.2	RM7C	54
**4th**	U	24.86667	V_RM7V	25.93333	RM7C_RM7V	49.33333
**5th**	RM7V	25.33333	RM7B	26.33333	RM7C_RM14C	47.23333
**6th**	RM14F	25.53333	RM7C_B	26.66667	RM14C_RM14F	45.46667
**7th**	RM7C_B	25.86667	RM7C_RM14F	26.8	RM14C_B	45.33333
**8th**	RM7C_RM7F	26.66667	RM7F_RM14F	27.53333	RM7C_RM14F	45.26667
**9th**	RM7F	26.86667	U	27.73333	RM14F	45.13333
**10th**	B	27.13333	V_RM14V	27.93333	RM14C_RM7V	44.93333
	**MSE**		**RMSE**		**RMSLE**	
	**Feature Setup**	**Friedman Score**	**Feature Setup**	**Friedman Score**	**Feature Setup**	**Friedman Score**
**1st**	V	21.93333	U	22.73333	U	17.6
**2nd**	B_RM7B	23.13333	V	23.13333	RM7C_RM14F	21.13333
**3rd**	RM7V	24	B_RM7B	23.66667	B_RM7B	22.53333
**4th**	V_RM7V	24.8	RM7B	24.46667	RM14F	22.73333
**5th**	RM7B	25.53333	RM14F	24.93333	V	23.2
**6th**	RM14F	26.73333	RM7V	25.4	RM7F	24.46667
**7th**	U	27.2	RM7C_RM7F	25.8	RM7C_RM7F	25.73333
**8th**	RM7C_RM7F	27.73333	RM7C_B	25.93333	RM7C_B	26.4
**9th**	B	27.86667	RM7C_RM14F	26.6	RM14C_RM7B	26.73333
**10th**	RM7C_B	27.93333	RM7F	27.2	B	26.86667

**Table A7:** Friedman results: feature setups—Shift 14.

	MAE		MAPE		R${}^{2}$	
	Feature Setup	Friedman Score	Feature Setup	Friedman Score	Feature Setup	Friedman Score
**1st**	RM7C_B	17.46667	RM7C_B	18.53333	RM14C	50.5
**2nd**	RM7C_V	21.53333	RM14C_B	22.2	RM7C	48.9
**3rd**	RM14C_B	22.26667	RM7C_V	22.93333	U	48.76667
**4th**	RM7C	22.26667	RM7F_RM14F	23.73333	RM14C_RM7F	48.16667
**5th**	U	23.73333	B_RM7V	24.2	RM14C_RM7B	46.83333
**6th**	B_RM7V	23.8	V	25.6	RM7C_RM7B	46.06667
**7th**	V	24.46667	RM7C_F	26.33333	RM7C_RM7F	45.76667
**8th**	RM7C_F	24.86667	V_RM14F	27.4	RM7C_RM14C	45.56667
**9th**	RM7B	25.86667	RM7C	27.66667	RM7F	43.83333
**10th**	RM14B	27.33333	RM7B	28.13333	RM14C_F	43.36667
	**MSE**		**RMSE**		**RMSLE**	
	**Feature Setup**	**Friedman Score**	**Feature Setup**	**Friedman Score**	**Feature Setup**	**Friedman Score**
**1st**	RM7C_B	18.26667	RM7C_B	15.86667	RM7C	13.8
**2nd**	RM7C_V	21.26667	RM7C	20.33333	RM7C_B	16
**3rd**	B_RM7V	21.6	RM14C_B	21.26667	RM14C_B	21.06667
**4th**	RM14C_B	23.86667	RM7C_V	21.4	U	22.33333
**5th**	V	25.06667	U	22.8	RM7C_F	24.13333
**6th**	RM7B	25.86667	RM7C_F	24	RM14B	24.33333
**7th**	RM7C	26.2	V	24.06667	B_RM7V	25.06667
**8th**	RM7C_F	26.26667	B_RM7V	24.46667	RM7C_V	26.06667
**9th**	RM7B_RM7F	26.33333	RM7B	26	V	26.26667
**10th**	RM7B_RM7V	26.66667	RM7C_RM14C	26.2	RM7B	26.46667

## References

[1] Basak S., Kar S., Saha S., Khaidem L., Dey S.R. (2019). Predicting the direction of stock market prices using tree-based classifiers. N. Am. J. Econ. Financ..

[2] Ren R., Wu D.D., Liu T. (2019). Forecasting Stock Market Movement Direction Using Sentiment Analysis and Support Vector Machine. IEEE Syst. J..

[3] Huang W., Nakamori Y., Wang S.Y. (2005). Forecasting stock market movement direction with support vector machine. Comput. Oper. Res..

[4] Zhong X., Enke D. (2019). Predicting the daily return direction of the stock market using hybrid machine learning algorithms. Financ. Innov..

[5] Abraham B., Ledolter J. (1983). Statistical Methods for Forecasting.

[6] Armstrong J.S., Collopy F.L. (1998). Integration of Statistical Methods and Judgment for Time Series Forecasting: Principles from Empirical Research. Forecast. Model. eJournal.

[7] Bontempi G., Ben Taieb S., Le Borgne Y.A. (2013). Machine Learning Strategies for Time Series Forecasting.

[8] Masini R.P., Medeiros M.C., Mendes E.F. (2021). Machine learning advances for time series forecasting. J. Econ. Surv..

[9] Cao L., Tay F. (2003). Support vector machine with adaptive parameters in financial time series forecasting. IEEE Trans. Neural Netw..

[10] Yang A., Li W., Yang X. (2019). Short-term electricity load forecasting based on feature selection and Least Squares Support Vector Machines. Knowl.-Based Syst..

[11] Sagheer A., Kotb M. (2019). Time series forecasting of petroleum production using deep LSTM recurrent networks. Neurocomputing.

[12] Zhao Z., Chen W., Wu X., Chen P.C.Y., Liu J. (2017). LSTM network: A deep learning approach for short-term traffic forecast. IET Intell. Transp. Syst..

[13] Graf R., Zhu S., Sivakumar B. (2019). Forecasting river water temperature time series using a wavelet–neural network hybrid modelling approach. J. Hydrol..

[14] Kurumatani K. (2020). Time series forecasting of agricultural product prices based on recurrent neural networks and its evaluation method. SN Appl. Sci..

[15] Khairalla M.A.E., Ning X., Al-Jallad N.T., El-Faroug M.O. (2018). Short-Term Forecasting for Energy Consumption through Stacking Heterogeneous Ensemble Learning Model. Energies.

[16] Alkandari M., Ahmad I. (2020). Solar power generation forecasting using ensemble approach based on deep learning and statistical methods. Appl. Comput. Inform..

[17] Liapis C.M., Karanikola A., Kotsiantis S.B. Energy Load Forecasting: Investigating Mid-Term Predictions with Ensemble Learners. Proceedings of the AIAI.

[18] Liapis C.M., Karanikola A.C., Kotsiantis S.B. An ensemble forecasting method using univariate time series COVID-19 data. Proceedings of the 24th Pan-Hellenic Conference on Informatics.

[19] Liapis C.M., Karanikola A., Kotsiantis S.B. (2021). A Multi-Method Survey on the Use of Sentiment Analysis in Multivariate Financial Time Series Forecasting. Entropy.

[20] Siami-Namini S., Tavakoli N., Namin A.S. A Comparison of ARIMA and LSTM in Forecasting Time Series. Proceedings of the 2018 17th IEEE International Conference on Machine Learning and Applications (ICMLA).

[21] Çıbıkdiken A., Karakoyun E. Comparison of ARIMA Time Series Model and LSTM Deep Learning Algorithm for Bitcoin Price Forecasting. Proceedings of the 13th multidisciplinary academic conference in Prague.

[22] Yamak P.T., Yujian L., Gadosey P.K. A Comparison between ARIMA, LSTM, and GRU for Time Series Forecasting. Proceedings of the ACAI.

[23] Maleki A., Nasseri S., Aminabad M.S., Hadi M. (2018). Comparison of ARIMA and NNAR Models for Forecasting Water Treatment Plant’s Influent Characteristics. KSCE J. Civ. Eng..

[24] Satrio C.B.A., Darmawan W., Nadia B.U., Hanafiah N. (2021). Time series analysis and forecasting of coronavirus disease in Indonesia using ARIMA model and PROPHET. Procedia Comput. Sci..

[25] Paliari I., Karanikola A., Kotsiantis S.B. A comparison of the optimized LSTM, XGBOOST and ARIMA in Time Series forecasting. Proceedings of the 2021 12th International Conference on Information, Intelligence, Systems & Applications (IISA).

[26] Zhang Y., Yang H.L., Cui H., Chen Q. (2019). Comparison of the Ability of ARIMA, WNN and SVM Models for Drought Forecasting in the Sanjiang Plain, China. Nat. Resour. Res..

[27] Tealab A. (2018). Time series forecasting using artificial neural networks methodologies: A systematic review. Future Comput. Inform. J..

[28] Sezer O.B., Gudelek M.U., Ozbayoglu A.M. (2020). Financial Time Series Forecasting with Deep Learning: A Systematic Literature Review: 2005–2019. arXiv.

[29] Lara-Benítez P., Carranza-García M., Santos J.C.R. (2021). An Experimental Review on Deep Learning Architectures for Time Series Forecasting. Int. J. Neural Syst..

[30] Karanikola A., Liapis C.M., Kotsiantis S., Tsihrintzis G.A., Virvou M., Jain L.C. (2022). A Comparison of Contemporary Methods on Univariate Time Series Forecasting. Advances in Machine Learning/Deep Learning-Based Technologies: Selected Papers in Honour of Professor Nikolaos G. Bourbakis—Volume 2.

[31] Wang K., Qi X., Liu H. (2019). A comparison of day-ahead photovoltaic power forecasting models based on deep learning neural network. Appl. Energy.

[32] Rao T., Srivastava S. Analyzing Stock Market Movements Using Twitter Sentiment Analysis. Proceedings of the International Conference on Advances in Social Networks Analysis and Mining.

[33] Nguyen T.H., Shirai K., Velcin J. (2015). Sentiment analysis on social media for stock movement prediction. Expert Syst. Appl..

[34] Kalyani J., Bharathi H.N., Jyothi R. (2016). Stock trend prediction using news sentiment analysis. arXiv.

[35] Shah D., Isah H., Zulkernine F.H. Predicting the Effects of News Sentiments on the Stock Market. Proceedings of the 2018 IEEE International Conference on Big Data (Big Data).

[36] Souma W., Vodenska I., Aoyama H. (2019). Enhanced news sentiment analysis using deep learning methods. J. Comput. Soc. Sci..

[37] Valle-Cruz D., Fernandez-Cortez V., Chau A.L., Sandoval-Almazán R. (2021). Does Twitter Affect Stock Market Decisions? Financial Sentiment Analysis During Pandemics: A Comparative Study of the H1N1 and the COVID-19 Periods. Cogn. Comput..

[38] Sharma V., Khemnar R.K., Kumari R.A., Mohan B.R. Time Series with Sentiment Analysis for Stock Price Prediction. Proceedings of the 2019 2nd International Conference on Intelligent Communication and Computational Techniques (ICCT).

[39] Pai P.F., Liu C. (2018). Predicting Vehicle Sales by Sentiment Analysis of Twitter Data and Stock Market Values. IEEE Access.

[40] Mohan S., Mullapudi S., Sammeta S., Vijayvergia P., Anastasiu D. Stock Price Prediction Using News Sentiment Analysis. Proceedings of the 2019 IEEE Fifth International Conference on Big Data Computing Service and Applications (BigDataService).

[41] Mehta P., Pandya S., Kotecha K. (2021). Harvesting social media sentiment analysis to enhance stock market prediction using deep learning. PeerJ Comput. Sci..

[42] Jin Z., Yang Y., Liu Y. (2019). Stock closing price prediction based on sentiment analysis and LSTM. Neural Comput. Appl..

[43] Wu S.H., Liu Y., Zou Z., Weng T.H. (2021). S_I_LSTM: Stock price prediction based on multiple data sources and sentiment analysis. Connect. Sci..

[44] Jing N., Wu Z., Wang H. (2021). A hybrid model integrating deep learning with investor sentiment analysis for stock price prediction. Expert Syst. Appl..

[45] Smailovic J., Grcar M., Lavra N., Znidarsic M. (2014). Stream-based active learning for sentiment analysis in the financial domain. Inf. Sci..

[46] Raju S.M., Tarif A.M. (2020). Real-Time Prediction of BITCOIN Price using Machine Learning Techniques and Public Sentiment Analysis. arXiv.

[47] Abraham J., Higdon D.W., Nelson J., Ibarra J. (2018). Cryptocurrency Price Prediction Using Tweet Volumes and Sentiment Analysis. SMU Data Sci. Rev..

[48] Valencia F., Gómez-Espinosa A., Valdés-Aguirre B. (2019). Price Movement Prediction of Cryptocurrencies Using Sentiment Analysis and Machine Learning. Entropy.

[49] Deb A., Lerman K., Ferrara E. (2018). Predicting Cyber Events by Leveraging Hacker Sentiment. Information.

[50] Masri S., Jia J., Li C., Zhou G., Lee M.C., Yan G., Wu J. (2019). Use of Twitter data to improve Zika virus surveillance in the United States during the 2016 epidemic. BMC Public Health.

[51] Chauhan P., Sharma N., Sikka G. (2021). The emergence of social media data and sentiment analysis in election prediction. J. Ambient. Intell. Humaniz. Comput..

[52] Tseng K.K., Lin R.F.Y., Zhou H., Kurniajaya K.J., Li Q. (2018). Price prediction of e-commerce products through Internet sentiment analysis. Electron. Commer. Res..

[53] Twintproject Twintproject/Twint: An Advanced Twitter Scraping & OSINT Tool. https://github.com/twintproject/twint.

[54] Van Rossum G. (2020). The Python Library Reference, Release 3.8.2.

[55] Bird S. (2004). NLTK: The Natural Language Toolkit. arXiv.

[56] Bird S., Klein E., Loper E. (2009). Natural Language Processing with Python.

[57] String—Common String Operations. https://docs.python.org/3/library/string.html.

[58] Simplified Text Processing. https://textblob.readthedocs.io/en/dev/.

[59] Hutto C.J., Gilbert E. VADER: A Parsimonious Rule-Based Model for Sentiment Analysis of Social Media Text. Proceedings of the International AAAI Conference on Web and Social Media.

[60] Araci D. (2019). FinBERT: Financial Sentiment Analysis with Pre-trained Language Models. arXiv.

[61] Devlin J., Chang M.W., Lee K., Toutanova K. (2019). BERT: Pre-training of Deep Bidirectional Transformers for Language Understanding. arXiv.

[62] ProsusAI ProsusAI/finBERT: Financial Sentiment Analysis with Bert. https://github.com/ProsusAI/finBERT.

[63] Malo P., Sinha A., Korhonen P.J., Wallenius J., Takala P. (2014). Good debt or bad debt: Detecting semantic orientations in economic texts. J. Assoc. Inf. Sci. Technol..

[64] timeseriesAI Timeseriesai/Tsai: Time Series Timeseries Deep Learning Machine Learning Pytorch FASTAI: State-of-the-Art Deep Learning Library for Time Series and Sequences in Pytorch/Fastai. https://github.com/timeseriesAI/tsai.

[65] Wang Z., Yan W., Oates T. Time series classification from scratch with deep neural networks: A strong baseline. Proceedings of the 2017 International Joint Conference on Neural Networks (IJCNN).

[66] Oguiza I. tsAI Models: FCNPlus. https://timeseriesai.github.io/tsai/models.fcnplus.html.

[67] Fawaz H.I., Lucas B., Forestier G., Pelletier C., Schmidt D.F., Weber J., Webb G.I., Idoumghar L., Muller P.A., Petitjean F. (2020). InceptionTime: Finding AlexNet for Time Series Classification. arXiv.

[68] Oguiza I. tsAI Models: InceptionTimePlus. https://timeseriesai.github.io/tsai/models.inceptiontimeplus.html.

[69] Oguiza I. tsAI Models: RNNS. https://timeseriesai.github.io/tsai/models.rnn.html.

[70] Hochreiter S., Schmidhuber J. (1997). Long Short-Term Memory. Neural Comput..

[71] Chung J., Gülçehre C., Cho K., Bengio Y. (2014). Empirical Evaluation of Gated Recurrent Neural Networks on Sequence Modeling. arXiv.

[72] Oguiza I. tsAI Models: RNN_FCN. https://timeseriesai.github.io/tsai/models.rnn_fcn.html.

[73] Karim F., Majumdar S., Darabi H., Chen S. (2018). LSTM Fully Convolutional Networks for Time Series Classification. IEEE Access.

[74] Elsayed N., Maida A., Bayoumi M.A. (2019). Deep Gated Recurrent and Convolutional Network Hybrid Model for Univariate Time Series Classification. arXiv.

[75] Oguiza I. tsAI Models: RNN_FCNPlus. https://timeseriesai.github.io/tsai/models.rnn_fcnplus.html.

[76] Zou X., Wang Z., Li Q., Sheng W. (2019). Integration of residual network and convolutional neural network along with various activation functions and global pooling for time series classification. Neurocomputing.

[77] Oguiza I. tsAI Models: ResNetPlus. https://timeseriesai.github.io/tsai/models.resnetplus.html.

[78] Bai S., Kolter J.Z., Koltun V. (2018). An Empirical Evaluation of Generic Convolutional and Recurrent Networks for Sequence Modeling. arXiv.

[79] Zerveas G., Jayaraman S., Patel D., Bhamidipaty A., Eickhoff C. A Transformer-based Framework for Multivariate Time Series Representation Learning. Proceedings of the 27th ACM SIGKDD Conference on Knowledge Discovery & Data Mining.

[80] Oguiza I. tsAI Models: TSTPlus. https://timeseriesai.github.io/tsai/models.tstplus.html.

[81] Oguiza I. tsAI Models: TSIT. https://timeseriesai.github.io/tsai/models.tsitplus.html.

[82] Oguiza I. tsAI Models: Transformermodel. https://timeseriesai.github.io/tsai/models.transformermodel.html.

[83] Fauvel K., Lin T., Masson V., Fromont E., Termier A. (2021). XCM: An Explainable Convolutional Neural Network for Multivariate Time Series Classification. arXiv.

[84] Oguiza I. tsAI Models: XCMPlus. https://timeseriesai.github.io/tsai/models.xcmplus.html.

[85] Rahimian E., Zabihi S., Atashzar S.F., Asif A., Mohammadi A. (2019). XceptionTime: A Novel Deep Architecture based on Depthwise Separable Convolutions for Hand Gesture Classification. arXiv.

[86] Oguiza I. tsAI Models: XceptionTimePlus. https://timeseriesai.github.io/tsai/models.xceptiontimeplus.html.

[87] Tang W., Long G., Liu L., Zhou T., Blumenstein M., Jiang J. (2022). Omni-Scale CNNs: A simple and effective kernel size configuration for time series classification. arXiv.

[88] Friedman M. (1937). The Use of Ranks to Avoid the Assumption of Normality Implicit in the Analysis of Variance. J. Am. Stat. Assoc..

[89] Dunn O.J. (1961). Multiple Comparisons among Means. J. Am. Stat. Assoc..

[90] Hodges J.L., Lehmann E.L. (1962). Rank Methods for Combination of Independent Experiments in Analysis of Variance. Ann. Math. Stat..

